# The complex role of IL-10 in malignant ascites: a review

**DOI:** 10.1007/s00262-023-03616-y

**Published:** 2024-01-27

**Authors:** Yue Huang, Kangni Zou, Heng Jiang, Zhengyu Li

**Affiliations:** 1 Department of Gynecology and Obstetrics, West China Second University Hospital, Sichuan University, Chengdu, 610041 People’s Republic of China; 2 Key Laboratory of Birth Defects and Related Diseases of Women and Children (Sichuan University), Ministry of Education, Chengdu, 610041 People’s Republic of China; 3 College of Medicine, University of Nebraska Medical Center, Omaha, NE USA

**Keywords:** Malignant ascites, Interleukin-10, Immunotherapy, Tumor microenvironment

## Abstract

The emergence of malignant ascites (MA) indicates poor prognoses in patients with ovarian, gastrointestinal, breast, and pancreatic cancer. Interleukin-10 (IL-10) is a pleiotropic cytokine with immunoregulatory effects in tumor microenvironment. The level of IL-10 in MA varied across cancer types and patients, influencing cancer progression and outcomes. Originating from various immune and cancer cells, IL-10 contributes to complex signaling pathways in MA. Systemic IL-10 administration, although the evidence of its efficacy on MA is limited, still emerges as a promising therapeutic strategy because it can increase CD8+ T cells cytotoxicity and invigorate exhausted CD8+ tumor infiltration lymphocytes (TILs) directly. IL-10 signaling blockade also demonstrates great potential when combined with other immunotherapies in MA treatment. We reviewed the levels, origins, and functions of IL-10 in malignant ascites and overviewed the current IL-10 signaling targeting therapies, aiming to provide insights for MA treatment.

**Supplementary Information:**

The online version contains supplementary material available at 10.1007/s00262-023-03616-y.

## Introduction

Malignant ascites (MA) is defined as the peritoneal fluid collection containing malignant cells in a cancer patient. Substantial amount of ascites can lead to abdominal swelling, nausea, early satiety, edema, pain, and even dyspnea, decreasing quality of life and usually indicating a unfavorable prognosis [1–3]. MA is commonly associated with primary abdomen or pelvic malignancies such as liver, colon, or ovarian cancers; it can also manifest in metastatic diseases. The precise molecular mechanisms underlying MA remain elusive, but increased vascular permeability and peritoneal lymphatic obstruction are recognized as key pathophysiological processes involving multiple cytokines. Notably, vascular endothelial growth factors (VEGF), a prominent vascular permeability factor, exhibit heightened expression in MA [4–7]. Additionally, other cytokines like tumor necrosis factor-$\alpha$, interleukin-6, interleukin-8, and interleukin-10 (IL-10) are elevated in the context of ovarian cancer (OC) patients with MA [8–10]. The intricate interplay of these cytokines may contribute to the impediment of fluid absorption and drainage, as tumor cells disseminate and proliferate within the peritoneal cavity, potentially obstructing lymphatic vessels [5, 11]. In addition to these primary mechanisms, neovascularization induced by matrix metalloproteinase (MMPs) and the retention of hypertonic fluids secreted by tumor may also play pivotal roles in the formation of MA [12–16] (Figure S1).

There is no clear guidance on managing MA, but the treatments mainly fall into either relieving symptoms or controlling the primary diseases. Intermittent paracentesis is the most widely used intervention for controlling MA clinically, and its modified technique, cell-free and concentrated ascites reinfusion therapy is applied in Japan, which is thought to avoid protein loss compared with conventional paracentesis [17]. However, paracentesis relieves symptoms temporarily, with the ascites rapidly reaccumulating, increasing the risks of rehospitalization and deteriorating the quality of life (QOL) [16]. Long-term drainage approaches include indwelling catheters, indwelling peritoneal ports, and peritoneovenous shunts (PVSs). Palliation rates of long-term drainage approaches achieve 97%, while their complication rates all exceed paracentesis, and PVSs had the highest complication rate (26–55%) [18]. Since the presence of MA usually indicates a more advanced disease status, targeting the primary diseases can be quite challenging. Intraperitoneal chemotherapy (i.p. chemotherapy) is considered to be effective for controlling MA, and the efficacy and safety of various agents have been investigated. Catumaxomab is a mouse/rat monoclonal antibody targeting epithelial cell-adhesion molecule (EpCAM) and has been approved for MA treatment by European Union in 2009 [19]. A phase II/III trial found that paracentesis plus catumaxomab could increase progress-free survival and improve QOL in patients with MA compared to paracentesis alone [20, 21]. A phase II clinical trial results demonstrated the safety and efficacy of the combination chemotherapy comprising of intravenous paclitaxel (PTX) and intraperitoneal S-1 in pancreatic cancer patients with MA [22]. Moreover, bevacizumab, a VEGF inhibitor, is considered as an option for recurrent OC patients with ascites, but with increased risk of GI perforation [23, 24]. A multicenter double-blind, placebo-controlled phase II trial explored intraperitoneally applied bevacizumab in patients with advanced gastrointestinal cancer and MA, and the results showed that bevacizumab was well tolerated but without efficacy in controlling ascites-related symptoms [25]. Hyperthermic intraperitoneal chemotherapy (HIPEC) is another i.p. chemotherapy technique which is thought to increase chemotherapy penetration and enhance cancer cells sensitivity to chemotherapy throughout the peritoneal cavity by heated solution delivery. It is recommended by the NCCN guidelines to use in FIGO stage III OC patients who undertook neoadjuvant chemotherapy (NACT) and interval debulking surgery (IDS) [23, 26]. Moreover, cytoreductive surgery with HIPEC is recommended for patients with gastric cancer and limited peritoneal metastases [27]. However, the application of HIPEC in colon cancer patients with peritoneal metastases remains controversial as several trials found the technique increased morbidity and mortality [28]. Both palliation therapies and methods targeting primary diseases have shown improvements in QOL, but did not demonstrate obvious advantages in survival of patients with MA. As a result, therapies with less complications and higher efficacy are warranted.

IL-10, a pleiotropic cytokine, plays a crucial role in both inflammation and immunity within tumor microenvironment. It is primarily produced by innate immune cells, including monocytes, dendritic cells (DCs), macrophages, and natural killer cells (NK cells), and adaptive immune cells such as CD4+ T cells, CD8+ T cells, Th17 cells, and B cells [29, 30]. Studies also indicate that tumor cells can secrete IL-10 [31, 32]. Cancer patients, in comparison with healthy people and those with benign tumors, exhibit elevated levels of IL-10 in both serum and ascites, with this correlating with advanced clinical staging and poor outcomes [33–36]. Notably, IL-10 in OC contributes to ascites-mediated apoptosis resistance and transcriptomic data identifying IL-10 in ascites as a robust indicator of poor outcomes for OC patients [37, 38]. Nevertheless, the precise role of IL-10 in oncogenesis remains controversial, with reported anti- and pro-tumor effects observed in various malignancies such as thyroid cancer, non-small cell lung cancer, breast cancer, and colorectal cancer [39–47]. IL-10 predominantly exerts its inhibitory function on macrophages, while its impact on T cells is complex, involving both the inhibition of T cell responses and the stimulation of CD8+ T cell proliferation [48–50]. Despite extensive research on MA, a comprehensive review focused on IL-10 in MA is notably absent. Consequently, this review aims to summarize the role of IL-10, spanning its levels to therapeutic targeting in MA, with the intent of providing evidence for the potential application of IL-10 in MA from bench to bedside.

### Levels of IL-10 in malignant ascites

Numerous studies have appraised IL-10 concentrations in diverse specimens obtained from cancer patients, consistently reporting elevated IL-10 levels in MA compared to non-malignant ascites (Table 1). The association between IL-10 levels in MA and patients’ survival outcomes remains controversial, with IL-10 not yet acknowledged as a prognostic biomarker for MA patients [10, 51–53]. Notably, a positive correlation between serum IL-10 levels and ascitic IL-10 concentrations in OC patients has been established, with significantly higher IL-10 levels detected in MA than in corresponding serum samples [10, 54]. Post-debulking surgery and/or chemotherapy interventions led to reduction in IL-10 levels in the in serum samples of OC patients [34, 55–57]. Furthermore, Antoneeva et al. revealed that IL-10 levels in the serum of OC patients peaked upon disease progression after NACT, suggesting its potential as a marker for predicting patients’ response after NACT [58]. Yigit et al. reported undetectable IL-10 levels in MA following NACT; interestingly, they also found minimal IL-10 levels in recurrent patients [52]. Explorations of IL-10 level change in MA after chemotherapy, using ID8 tumor-bearing mice, indicating a decreasing trend, though statistical significance was not achieved [59]. Additionally, Park et al. disclosed higher IL-10 levels in MA of metastatic gastric cancer (mGC) patients compared to healthy volunteers’ body fluids. Elevated levels of at least two cytokines (VEGF-A, IL-10, or TGF-beta) in MA correlated with shorter overall survival (OS) in mGC patients, yet IL-10 alone did not emerge as an independent risk factor for shorter OS in mGC patients [60]. Atta et al. demonstrated elevated IL-10 levels in ascites of patients with hepatocellular carcinoma (HCC) compared to those with cirrhosis [61]. Given the disparate outcomes and limited sample sizes of prior studies, a comprehensive evaluation of IL-10 levels in MA before and after treatment, particularly within a sizable population, is warranted. Despite the potential of IL-10 level changes during treatment to shed light on the prognostic value of IL-10 in MA, a dearth of data currently exists on this subject.

**Table 1:** IL-10 levels of MA in previous reported studies

Study	Patients	IL-10 (pg/mL)	Results
Gotlieb [33]	35 (Intraperitoneal malignancies: 11 serous, 4 endometrioid, 3 mucinous, 1 undifferentiated, 1 clear cell ovarian cancer, 1 dysgerminoma, 4 mixed mesodermal tumors, 1 uterine leiomyoma, 1 transitional carcinoma, 3 peritoneal adenocarcinomas, 1 mesothelioma, 1 Krukenberg tumor)	542 ± 77	IL-10 in peritoneal fluids (*n* = 63) obtained during surgery for benign gynecological conditions (34.2 ± 7.5 pg/mL) IL-10 levels were also elevated in the serum of patients with intra-abdominal cancer (1353 ± 906, *n* = 8)
Santin [62]	28 FIGO Stage III-IVA ovarian cancer, treated by radical surgical tumor debulking and adjuvant chemotherapy	165 ± 137 (50–556)	Serum of normal controls: 12 ± 5 pg/mL (8–23, *n* = 10); IL-10 levels were significantly higher in ascitic fluid than in plasma
Nowak [35]	37 EOC patients	258.1 ± 38.9 102.5 ± 21.8 (Stage I/II) *n* = 9 302.5 ± 46.4 (Stage III/IV) *n* = 28	AUC-ROC showed good predictive values for IL-6 (0.87), IL-10 (0.836) and IL-8 (0.797)
Matte [38]	38 EOC patients early (stage I/II; *n* = 12) and advanced (stage III/IV; *n* = 26)	24(0–488)	Patients with high levels of IL-10 in ascites (> 24 pg/mL) also showed shorter PFS with a median PFS of 14 months for high IL-10 levels versus 36 months for patients with low IL-10
Zhu [63]	16 EOC patients	99.81 ± 71.67	IL-10 was significantly higher in the ascites of EOC patients than that in the blood serum of the healthy subjects (7.23 ± 7.26 pg/mL)
Giuntoli [54]	22 EOC patients FIGO III/IV	137.85 ± 25.52	Plasma 17.60 ± 5.92
Gening [64]	11 primary OC patients	411,661 (355,644–540,201)	Plasma 8971 (4500–16,407)
Sipak-Szmigiel [10]	38 OC patients	128.80 (36.71–252.38)	Serous cyst 18.15 (4.60–41.70) *n* = 54; endometrioid 53.00 (14.70–93.20) *n* = 43; Serum 11.30 (4.60–21.00)
Zhou [32]	32 OC patients (8 FIGO II; 24 FIGO III/IV)	140.43 (111.72–209.09)	IL-10 concentration in ascitic fluid > serum. IL-10 level in serum from OC patients > benign tumor or normal volunteers IL-10 level in the ascites of stage III/IV > stage II patients
Yigit [52]	38 OC patients (3 FIGO I; 35 FIGO III/IV) untreated primary OC	0 (0–28); 54 (0–4266)	In advanced-stage tumor ascites, IL-10 in untreated primary disease > after neo-adjuvant treatment. In the latter group (*n* = 5), IL-10 was not detectable in any of the samples. IL-10 concentrations in untreated primary tumor ascites > in ascites obtained at recurrence
Naldini [65]	12 (Stage IV of OC)	195 ± 54	
Cândido [66]	44 EOC	No optimal cytoreduction: 178.5 (61.6–561.3) Optimal cytoreduction: 46.60 (3.8–255.3)	The IL-10 levels in the peritoneal fluid were higher in patients with stage III/IV ovarian cancer Patients with unsatisfactory cytoreduction had higher levels of IL-10 in the peritoneal fluid
Sasada [67]	6 gastric cancer, 1 pancreatic cancer	518 pg/mL 53 pg/mL 1197 pg/mL 221 pg/mL 76 pg/mL 19 pg/mL 594 pg/mL	
Atta [61]	25 healthy control vs 20 patients with hepatic lesions	5.57 ± 5.88 pg/mL in healthy patients; 9.91 ± 10.44 pg/mL in patients with hepatic lesions	IL-10 level was significantly higher in group II than in group I. There was a high significant increase ascitic fluid IL-10 level of patients with hepatic lesions compared to the serum level of the same patients and normal individuals

Most studies focus on cytokines in MA as prognostic markers. IL-6, IL-10, TNF-α, IFN-γ, TNF-α have been substantiated as directly linked to poor outcomes in epithelial ovarian cancer (EOC) patients. [51, 66] However, research into the interplay among these cytokines remains limited. Reinartz et al. established transcriptome-derived datasets from ascites cells of 29 EOC patients, revealing that IL-10, IL-6, and leukemia inhibitory factor (LIF) activate downstream STAT3 signaling pathway, and these three cytokines are associated with early relapse. IL-10 and IL-6 played pivotal yet insufficient roles in inducing the proliferation of Myeloid-derived suppressor cells (MDSCs) [68]. Additionally, a robust association was observed between high IL-10 levels and elevated Arachidonic acid (AA) level [37]. Soluble human leukocyte antigen-G (sHLA-G) and IL-10 were also deemed functionally related [10, 53]. Certain tumor cells were found to produce IL-10, thereby upregulating the expression of HLA-G, which, in turn, induced immune cells to produce IL-10, IL-4 and IL-3 [69]. Notably, limited evidence exists regarding the association between IL-10 and other cytokines, necessitating further studies to explore these correlations.

## IL-10-producing cells and the possible functions

### Myeloid cells

Myeloid cells, derived from hematopoietic stem cells in the bone marrow, comprise granulocytes and monocytes, exerting intricate roles in the tumor microenvironment [70, 71]. Inflammation is closely linked to the cancer progression, with neutrophils considered a source of IL-10. In the context of OC, infiltrated neutrophils secrete various cytokines, including IL-2, IL-6, IL-10, TNF-α, and VEGF, promoting cancer progression in the tumor microenvironment [6]. Hart et al. identified a CD11b+ population of MDSCs in OC, which significantly produces IL-10. IL-10, along with other immunosuppressive molecules, induces myeloid maturation, activates MDSCs, alters T cells phenotypes, and reduces their IFN-γ production [72]. Subsequent studies revealed that the CX3CR1-negative subset is the primary source of IL-10 in early oncogenesis, while the positive subset becomes dominant in later stages [73]. IL-10 neutralization in ascites reverses the immunosuppressive effects of MDSCs, improving survival and alleviating tumor burden in OC patients [74]. Additionally, IL-10, IL-6, and STAT3 activation in OC ascites induces MDSCs accumulation, inhibiting autologous T cell proliferation and effector function [68].

Dendritic cells (DCs), as antigen-presenting cells, act as messengers between innate and adaptive immune responses. High IL-10 levels are secreted by immature DCs accumulating around tumor foci [75]. Plasmacytoid DCs (pDCs), a subset plasma cell morphology, migrate to OC microenvironment due to OC cells secreting CXCR4/SDF-1, upregulating VLA-5 expression [76]. Precursor pDCs in OC microenvironment induce regulatory T cells (Tregs), suppressing immune function by producing IL-10 [77]. Furthermore, DCs isolated from OC patients’ ascites induce IL-10 production in CD3+ CD4+ T cells [78].

Tumor-associated macrophages (TAMs) are another source of IL-10 in MA [79]. Contrary to immunosuppressive aspects, IL-10 secreted by TAMs induces Tregs by activating Foxp3 expression, leading to tumor progression [63]. IL-10, coupled with N-acetylaspartate (NAA) released by OC cells, induces glutamine synthetase (GS) expression in macrophages which acquired M2-like phenotype in OC ascites [80].

**Fig. 1:**
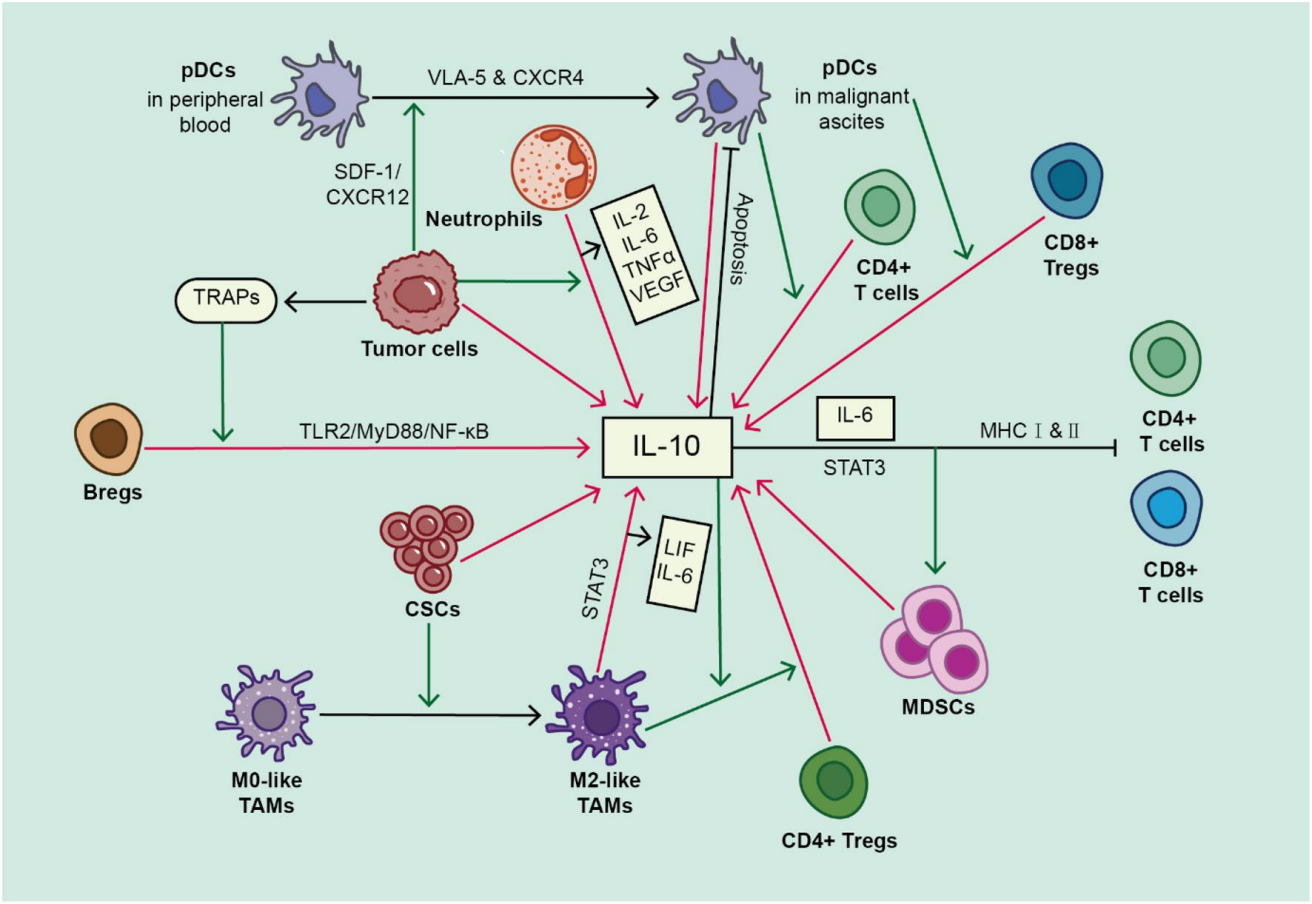
IL-10-producing cells and biological effects after IL-10 stimulation. Many cells in MA can produce IL-10, such as myeloid cells (including neutrophils, MDSCs, DCs and TAMs, etc.), lymphoid cells including T cells and B cells, and cancer cells. IL-10 in MA, in turn, can induce the maturation and activation of MDSCs, alter the phenotypes of T cells and stimulate IL-10 secretion in T cells and TAMs. The red arrows signal the IL-10-secreting process and the green arrows signal the promotion and proliferation process. Bregs, regulatory B cells. CSCs, cancer stem cells. MDSCs, myeloid-derived suppressor cells. pDCs, plasmacytoid dendritic cells. TAMs, tumor-associated macrophages. TRAPs, tumor cell-released autophagosomes. Tregs, regulatory T cells

In MA, a monocyte subset could produce IL-10, contributing to antitumor immune response by inhibiting T cell proliferation and cytokine production [14]. MA of OC patient induces strong IL-10 production and decreases INF-γ and IL-2 in resting and activated peripheral blood mononuclear cells (PBMCs), suggesting that OC MA favored Th2 inhibitory immune response [65]. Freedman et al. identified a subset of CD14+ HLA-DR- monocytes secreting IL-10 with suppressive activity against effector T cells [81] (Fig. 1).

### Lymphoid cells

Lymphoid cells, predominant in the lymph system, include NK cells, T cells, and B cells, playing crucial roles in immune response. T cells and B cells both produce IL-10 in MA. Tregs are a significant source of IL-10 in MA, inducing inhibitory receptor expression on both CD4+ T cells and CD8+ T cells along with IL-35 [67, 82]. Regulatory B cells (Bregs), a subset restraining excessive inflammatory responses, also produce IL-10 [83]. Several studies indicate increased IL-10-producing Bregs correlate with poor prognosis in gastric cancer patients. Evaluated tumor-infiltrating Bregs are associated with increased Tregs in patients with gastric cancer, OC, and tongue squamous cell carcinoma, indicating potential interactions between Tregs and Breg in immune evasion [84–87]. Zhou et al. isolated tumor cells-released autophagosomes (TRAPs) from malignant effusion and ascites, demonstrating TRAPs induce Bregs differentiation through the TLR2/MyD88/NF-κB signaling pathway, inhibiting CD4+ T cells and CD8+ T cells through IL-10 secretion [88]. The levels and functions of Bregs in MA require further exploration (Fig. 1).

### Cancer cells

Whether cancer cells could produce IL-10 is controversial. Gotlieb et al. reported undetectable levels of IL-10 in five OC cell lines (CAOV-3, SKOV-3, CC194, OC222, and OC 494) [33]. However, Berger et al. did not detect IL-10 in SKOV-3, CAOV-3, and OAW42 cell lines, but they found IL-10 expression in OVCAR-3 cell line [89]. Carr et al. found significant IL-10 production only in SW626 among several OC cell lines (including CAOV-3, CAOV-4, ES-2, OV-90, OVCAR-3, SKOV-3, TOV-21G, TOV-112D, TTB-6, COV413) [90]. Zhou et al. identified IL-10 production in SKOV-3, CAOV-3, and 3AO cell lines [32]. Nowak et al. isolated OC cells from patients’ tumor tissue and found both unstimulated and LPS-stimulated OC cells produce high levels of IL-10, with the FIGO staging correlated with IL-10 levels, while cells from benign ovarian tumors and normal tissue produced low IL-10 levels [91]. Recent studies identified cancer stem cells as a possible source of IL-10. Gening et al. found a strongly positive relationship between stem-like tumor cells (CD45-CD44+CD133-) and IL-10 levels in MA [64]. Raghavan et al. established a hanging drop spheroid model and found that hetero-spheroids containing cancer stem cells and M2-macrophages (CSC/M2 hetero-spheroids) could secrete IL-10, indicating CSCs from MA can produce IL-10 under M2-macrophage interaction. [92] Beyond OC cells, IL-10 has been found in several human carcinoma cell lines and cells isolated directly from tumor tissues, with pancreatic cancer cell-derived IL-10 suppressing CTL lytic function of T cells [93–95]. The metastatic ability of EOC cells correlates with IL-10 and TGF-β in ascites, associated with altered MMP-2 and TIMP-2 levels [96] (Fig. 1).

## Possible IL-10 signaling pathways in malignant ascites

The IL-10 signaling pathway is presumed to play a role in the development of immunosuppressive malignant ascites within the tumor microenvironment. Nevertheless, there is a scarcity of studies focusing on the downstream reactions of IL-10. A comprehensive understanding of IL-10 signal transduction in the development of MA can contribute to the integration of immunotherapeutic strategies. The receptor for IL-10 forms a functional complex consisting of two ligand-binding subunits, IL-10 R1 and IL-10 R2. Ligand engagement and binding are primarily facilitated by IL-10 R1, which exhibits high affinity, while IL-10R2 is responsible for transducing subsequent signals. In addition to being expressed on immune cells, Rabinovich et al. observed elevated IL-10 receptor expression on both OC tumor tissue and cells [31]. At the cell membrane surface, IL-10 binding to IL-10R1 and IL-10R2 activates Janus Kinase 1 (JAK1) and Tyrosine Kinase 2 (TYK2), phosphorylating the receptor complexes Y446 and Y496 located at the cytoplasmic tail. Subsequently, Signal Transducer and Activator of Transcription 3 (STAT3), a key transcription factor downstream of the IL-10 signaling pathway, is assembled and phosphorylated. It then translocates into the nucleus, binding to the promoter elements of relevant target genes and ultimately participating in the expression of pro-tumor and anti-apoptotic genes such as BCL2, TWIST, thereby exerting tumor-promoting effects [97–99]. Notably, IL-10 can also phosphorylate STAT1 and STAT5, perpetuating downstream signaling [100, 101]. Apart from the JAK1-STAT3 cascade, IL-10 is hypothesized to boost cancer stemness through the JAK1-STAT1-NF-$\kappa$ B pathway, thereby promoting tumor progression and metastasis [102]. However, contradictory findings exist, as some studies suggest that IL-10-mediated STAT1 activation induces pro-apoptotic and anti-proliferative effects in macrophages pre-treated with IFN-γ or IFN-α, contradicting IL-10-mediated STAT3 activation. The mechanism and switches of IL-10-mediated STAT pathway in MA remain unclear, with ongoing investigations into the involved mechanisms [103]. Additionally, Zhou et al. demonstrated that IL-10 could activate the Phosphatidylinositol 3-Kinase (PI3K) -AKT pathway, thereby regulating cell apoptosis [104]. Furthermore, Reitamo et al. discovered that under the influence of IL-10, the expression of MMPs, associated with neovascularization in MA, increased, suggesting a potential mechanisms by which IL-10 contributes to the formation of MA [105] (Figure S2).

## IL-10 as a prospective therapeutic target in malignant ascites

To harness IL-10 signaling, both recombinant IL-10 protein and the blockade of IL-10 and/or IL-10R are being explored for the MA treatment [106, 107]. The potential anti-tumor effects of IL-10 involve upregulating CD8+ T cells cytotoxicity, thereby reducing macrophage infiltration, increasing NK cell infiltration, resisting angiogenesis, and stabilizing intrinsic apoptosis [108]. Guo et al. demonstrated that a half-life-extended IL-10/Fc fusion protein could directly invigorate terminally exhausted CD8+ tumor-infiltrating lymphocytes (TILs) via oxidative phosphorylation and the process is in absence of the progenitor cells, making it a promising complementary therapy to immune checkpoint blockade in the treatment of patients with poor or none tumor infiltration of progenitor-exhausted CD8+ TILs [109]. Consequently, pegilodecakin, a pegylated form of recombinant IL-10 sustaining high concentration, has entered clinical trials. In a phase 1/1b trial (NCT02009449), one ovarian cancer patient and 22 pancreatic cancer patients were recruited to evaluate the safety and efficacy of pegilodecakin (pegylated IL-10) as monotherapy, and the results showed that patients did not have obvious clinical responses [110]. The result of SEQUOIA (NCT02923921), a phase 3 study evaluating pegilodecakin combined with folinic acid, fluorouracil, and oxaliplatin (FOLFOX) in patients with metastasis pancreatic ductal adenocarcinoma (PDAC), demonstrated that the addition of pegilodecakin did not increase efficacy of FOLFOX [111]. Recombinant IL-10 protein exerted promising anti-tumor effects on solid tumors such as renal cell carcinoma and melanoma, but ideal therapeutic effects did not be observed on patients with MA-associated cancer types such as ovarian cancer and pancreatic cancer, and the agent’s direct efficacy on MA remains unknown, impeding its further clinical application [107]. The complex tumor microenvironment and small sample size may account for these limited effects. Future research should specifically investigate recombinant IL-10 protein efficacy on MA.

Blocking IL-10 signaling also presents an immunotherapy strategy due to IL-10’s tumor immunosuppressive effect. Direct blockade via anti-IL-10 antibodies or indirectly use of anti-IL-10R antibodies can interfere with IL-10 function. IL-10/IL-10R neutralization has demonstrated more anti-tumor effects when combined with other immunotherapeutic strategies [112, 113]. Lamichhane et al. found that the combination of PD-1 blocking and IL-10 (R) neutralization activated more T and B cells, decreasing MDSCs in the ascites of ID8 tumor-bearing mice, leading to tumor burden alleviation and survival prolongation [114]. Adams et al. engineered a tumor vaccine by isolating ascites-derived monocytes from ID8 tumor-bearing mice and stimulating with Toll-like receptor (TLR) 4 lipopolysaccharide, TLR 9 CpG-oligonucleotides, and IL-10R antibody. The vaccine suppressed tumor growth and ascites generation in mice, and the proposed mechanism was macrophage activation and increased T cell response [115]. Additionally, IL-10 antibody enhanced the efficacy of OK-432’s locoregional immunotherapy for mouse breast cancer ascites [94]. Hu et al. showed that arsenic trioxide exerted a potential therapeutic response by decreasing IL-10 and TGF-β levels in TILs from ascites of gastric cancer patients, with the proportion of CD4+CD25+CD127-Tregs decreasing and the CD8+ T cells frequency and IFN-γ level increasing [116].

Furthermore, Martincuks et al. found that PARP inhibitors induced their therapy resistance against OC by activating STAT3 pathway, which closely related to the downstream pathway of IL-10. Thus, neutralization of IL-10/IL-10R in combination with PARP inhibitors could reduce therapeutic resistance [117]. Chimeric antigen receptor-T cell (CAR-T) therapy is defined as administrating reengineered chimeric T cells that can recognize tumor cells from cancer patients, enhancing T cell proliferation and reducing apoptosis. CAR-T therapy is different with conventional therapies as it can directly bypass the processing and presentation of tumor antigens by DCs, so it can avoid part of the immunosuppressive effect in the tumor microenvironment. In vitro experiments found that mesothelin CAR-T cells reacted with SKOV-3 cells, promoting the secretion of granzyme B and IFN-γ, which have important roles in producing cytotoxicity and killing OC cells. Blocking IL-10 signaling pathway can further increase granzyme B and IFN-γ levels. Therefore, blocking IL-10 signaling pathway has the potential to reverse the immunosuppressive effect in MA and enable stronger cytotoxicity produced by mesothelin CAR-T therapy [118, 119].

**Fig. 2:**
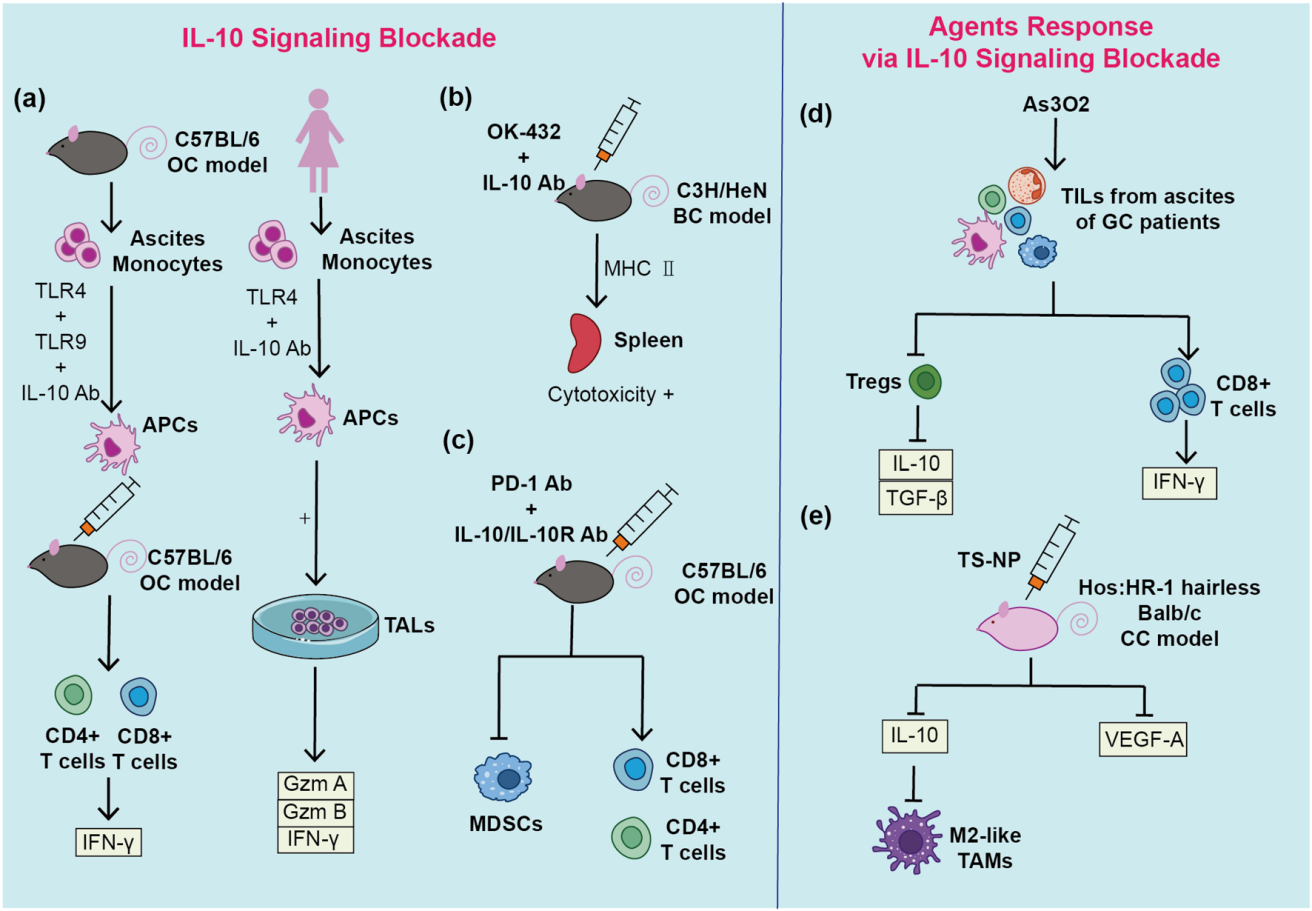
Therapeutic approaches mediated by IL-10 signaling blockade. APCs, antigen-presenting cells. GC, gastric cancer. IL-10 Ab, IL-10 antibody. IL-10R Ab, IL-10 receptor antibody. PD-1 Ab, PD-1 antibody. MDSCs, myeloid-derived suppressor cells. TALs, tumor-associated lymphocytes. TAMs, tumor-associated macrophages. TS-NP, α-Tocopheryl succinate nanoparticles

In addition to conventional therapies, emerging medical materials show promise in cancer treatments. Methotrexate-loaded gold nanoparticles (MTX-AuNP) inhibited ascites formation in mice with LL2 ascites tumors [120]. Silver nanoparticles (AgNPs) decreased ascites fluid volume in a lymphoma ascites mice model [121]. α-Tocopheryl succinate nanoparticles (TS-NP) prevented peritoneal dissemination, reducing tumor nodules, tumor weights, and ascites volume via VEGF-A and IL-10 levels decreasing and M2-like TAMs polarization decreasing [122]. Except for nanoparticles, peptide hydrogels stand for another promising drug-loading candidate. Dai et al. created a melittin hydrogel loaded with KN93 (CAMKII inhibitor), resulting in decreased ascites volume and reprogramming the ascites microenvironment in mice with hepatoma and MA [123]. Shamskhou et al. established a hyaluronan and heparin-based system delivery recombinant IL-10 for the treatment of lung fibrosis in vivo [124]. The above results indicate that it is realistic and pragmatic for novel materials like hydrogel system to load with IL-10 protein or IL-10/IL-10R antibody, enhancing their efficacy of immunoregulatory function in MA (Fig. 2).

IL-10 is a pleiotropic cytokine exerting both immunosuppression and immunostimulatory effects within the cancer microenvironment. The levels of IL-10 in MA exhibit considerable variability across different cancer types and even among patients with the same cancer type. Notably, IL-10 levels surpass those found in non-malignant ascites and exceed the serum IL-10 levels within the same patient. Several studies have proposed its potential as a marker for predicting patients' response following neoadjuvant chemotherapy. The cellular origins of IL-10 in MA are diverse, encompassing neutrophils, MDSCs, DCs, TAMs, T cells, B cells, and even cancer cells. Functionally, IL-10, secreted by MA cells, elicits multifaceted effects such as phenotypic alterations and the regulation of cytokines secretion. It is hypothesized that IL-10 could phosphorylate and activate STAT1, STAT3, STAT5, and PI3K pathways, exerting apoptosis regulation and tumor progression functions on immune cells. Systemic administration of IL-10 holds promise as an immunotherapy, as it can enhance CD8+ T cell cytotoxicity and invigorate terminally exhausted CD8+ TILs. Despite the therapeutic efficacy demonstrated by PEGylated human IL-10 (pegilodecakin) in certain solid tumors like renal cell carcinoma and melanoma, its effects on patients with MA remain unclear. Strategies including the blockade of IL-10 signaling, driven by its immunosuppressive effects, have shown therapeutic promise in both in vitro and in vivo experiments, particularly when combined with other immunotherapies. Effectively harnessing IL-10 pleiotropy is challenging yet essential in the treatment of MA. Further research is warranted in the following directions: (1) Explore cytokines interacting with IL-10 in MA to elucidate the cytokine network in microenvironment of MA. (2) Investigate the IL-10 signaling pathway, with a focus on the STAT1/STAT3 switches in MA. (3) Evaluate the therapeutic effects of recombinant IL-10 protein on patients with MA. (4) Develop appropriate IL-10 blocking agents and delivery systems, assessing their efficacy and safety in preclinical trials.

### Supplementary Information

Below is the link to the electronic supplementary material.

Supplementary file1 (DOCX 333 KB)

## Data Availability

N/A.
