# Supplementary material for: The complex role of IL-10 in malignant ascites: a review
Source: Cancer Immunol Immunother. 2024 Jan 27;73(2):32. doi: 10.1007/s00262-023-03616-y (PMC10821842; doi:10.1007/s00262-023-03616-y)
Supplement: Supplementary file 1 — Supplementary file1 (DOCX 333 KB) [file 262_2023_3616_MOESM1_ESM.docx]

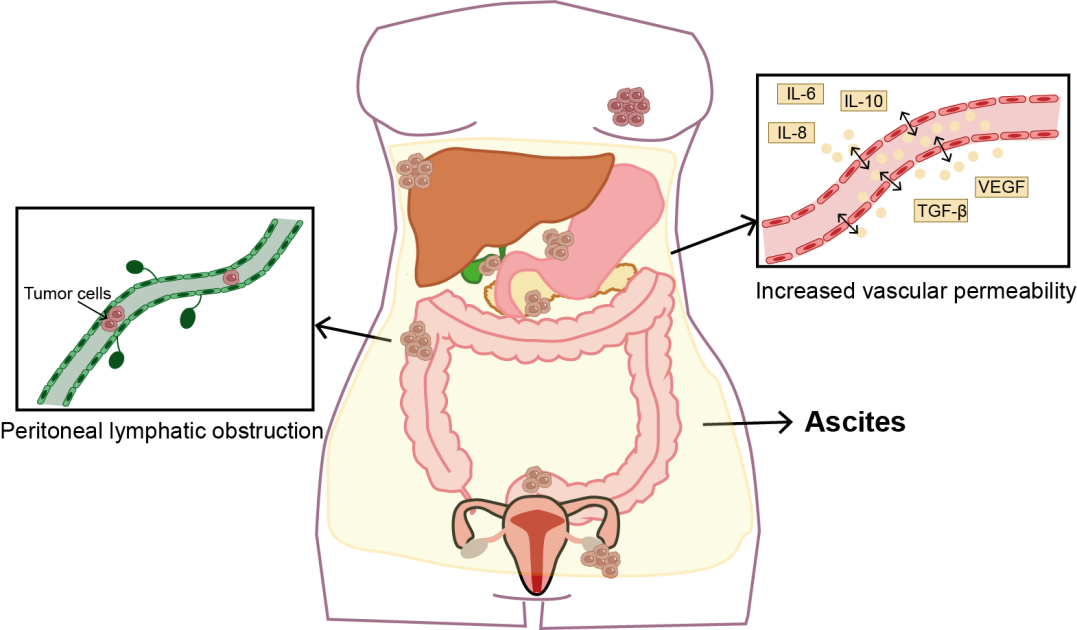


**Figure 1** Composition of malignant ascites. The formation of malignant ascites is associated with the increased permeability and peritoneal lymphatic obstruction. The permeability of endothelial cells is increased through the interaction between the growth factor, VEGF, and cytokines like IL-6, IL-8, IL-10. The dissemination and growth of the tumor cells obstruct the lymphatic vessels. Then, as an intraperitoneal cycle, malignant ascites can transfer downward by gravity and upward by respiration.


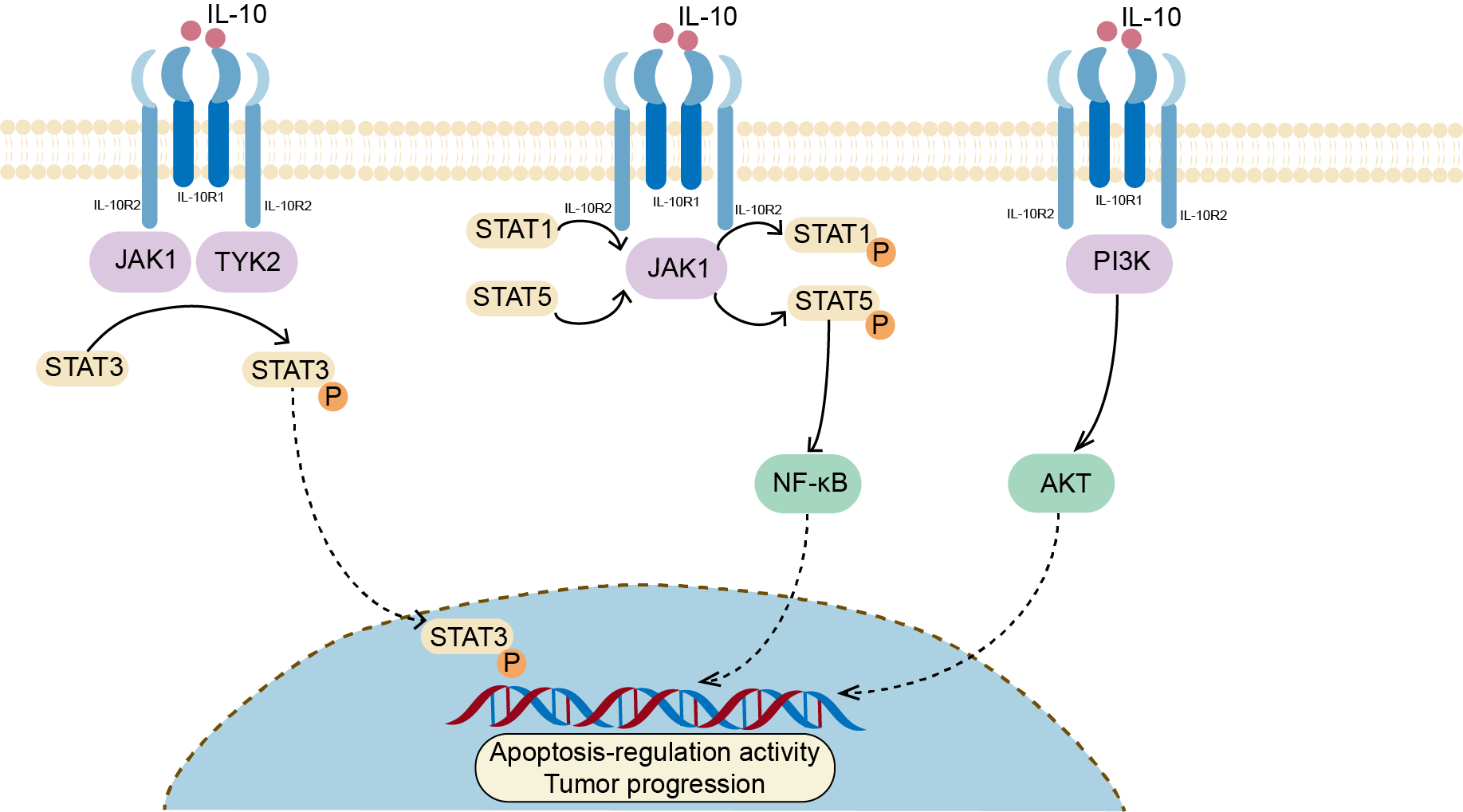


**Figure 3** Downstream signaling cascades of IL-10. The functional receptor complex, IL-10 receptor, comprising two subunits each of IL-10 R1 and IL-10 R2. IL-10 binds to IL-10R, which activates Jak1 and Tyk2 respectively, and phosphorylates the receptor complex. STAT1, STAT3, and STAT5 were shown to participate in the expression of apoptosis-regulation and tumor progression genes. Other cascade, such as PI3K-AKT pathway, is also proved to be involved in the transcription regulation of IL-10.
